# Editorial: The gut-skin-brain axis in human health and disease

**DOI:** 10.3389/fnut.2023.1155614

**Published:** 2023-02-16

**Authors:** Anita Ferraretto, Elena Donetti, Jaime García-Mena, Gustavo Pacheco-López

**Affiliations:** ^1^Department of Biomedical Sciences for Health, Università degli Studi di Milano, Milan, Italy; ^2^Centro de Investigación y de Estudios Avanzados del Instituto Politécnico Nacional (Cinvestav), Departamento de Genética y Biología Molecular, Mexico, Mexico; ^3^Health Sciences Department, Metropolitan Autonomous University (UAM), Lerma, Mexico

**Keywords:** food components, skin diseases, gut diseases, brain pathologies, impaired homeostasis

In the last decade, the gut-skin-brain axis has emerged as a research field offering more and more evidence on the way foods and intestinal microbiota can affect the development of intestinal/skin diseases (1), and neurological-psychiatric-psychological disorders (2). The possibility to prevent and/or treat these pathological conditions by modulating gut microbiota and/or food components would allow to diminish their prevalence, save the use of drugs, and understand the involved mechanisms. In this Research Topic, two *Original Research*, one *Hypothesis and Theory*, and one *Perspective* have addressed this issue by studying possible microbiota involvement and modulation in the case of psoriasis, epilepsy, depression, and a possible correlation with environmental exposure to glyphosate and glyphosate-based herbicides with neuropsychiatric conditions. In all these publications, diet, probiotics, and lifestyle changes represent also valid support for ameliorating health conditions.

The original research by Sun et al. considered two physiological body barriers, i.e. skin and gut, and investigated the relationship between intestinal microbiota and psoriasis development with an epidemiologic analysis and an experimental approach. In psoriatic patients, the incidence of gastrointestinal discomfort symptoms was significantly higher than in healthy subjects and the gut microbiota was improved when patients received therapy of oral acitretin and narrow-band ultraviolet B, with an evident decrease in their psoriasis area and severity index (PASI) score compared to untreated subjects. The transplantation of fecal microbiota from psoriatic patients or healthy controls into mice after the application of imiquimod cream confirmed that psoriatic microbes delayed recovery of psoriasiform dermatitis and induced a lesser decrease of IL-17A, a key proinflammatory psoriatic cytokine, thus validating the epidemiologic data. This study represents an excellent example of the translational integration of preclinical and clinical investigations and helps in better defining the complex association between dysbiosis and psoriasis with the final aim of developing microbiome-based therapeutic options for dermatologists.

The connection between the gut and brain has been investigated by Wang et al. for epilepsy, a disease imposing low quality of life for millions of people. The hypothesis of a possible gut dysbiosis has been considered by the authors based on the published studies about the regulating role of bacteria in neuroinflammation and oxidative stress. Under this perspective, the administration of probiotics, *Bifidobacterium* and *Lactobacillus* strains, prebiotics, inulin, and synbiotics, the combination of bacteria and inulin, were administered to kainic acid-induced epileptic rats, and both clinical manifestations and biochemical markers of the supposed involved mechanisms were collected. This pre-clinical results obtained show a decreased frequency and duration of seizures, an extended latency period with a major effect due to synbiotics, together with an improvement in cognitive impairment. The biochemical markers of lipid peroxidation, DNA damage, diminished total antioxidant ability, inflammation, and the release of IL-1β, IL-6, and TNF-α, confirm the authors' hypothesis and pave the way for finding new therapeutic agents.

Ghannoum et al. provide a *Hypothesis and Theory* contribution shedding light on the potential link between the gut microbiome and depression, exacerbated by the COVID-19 pandemic. The authors provided clinical evidence suggesting that integrative management of depression may also include microbiome modulation. Despite this issue deserves further data to be fully comprised, the possibility to help people to understand the causes of their mental problems and to avoid the use of antidepressant drugs, often without real positive effect or coupled with strong side effects and costs, is worth to be investigated.

In the *Perspective* opinion made by Barnett et al., the reader enlightens by the descriptive evidence of how glyphosate, which was designed to combat threatening weeds competing for nutrients and resources for crops production, induces alterations of the gut microbiota diversity, associating with neuropsychiatric conditions like anxiety, depression, autism, and important maladies like Parkinson and Alzheimer's disease. The discussion of the topic walks through a narrative describing how sub-toxic exposure to glyphosate and glyphosate-based herbicides is reported to perturb the abundance of important species in the microbial intestinal biome, which include commensals like *Lactobacillus* spp., *Bacteroides* spp, *Bifidobacterium* spp., *Butyricoccus* spp., *Clostridium* spp., and members of the family Ruminococcaceae, among others. The changes in the abundance of these bacterial species are the results of their susceptibility to the antimicrobial properties of glyphosate and result in the reduction of important microbial metabolites many of which are neurotransmitters, necessary for mental health and wellbeing acting through the gut-brain-microbiome axis. On the other hand, there is a selection of glyphosate-resistant microbes with the potential to increase the production of pro-inflammatory cytokines and reactive oxygen species which may result in dysfunctional hypothalamic-pituitary-adrenal axis activation, with deleterious consequences in neuronal homeostasis. The authors finish with a discussion of the benefits that the use of glyphosate and glyphosate-resistant plants brought to crop production in the world, which permits us to understand the need for the development of alternative methods for weed control.

In conclusion, the published works evidence that perturbations of the gut microbiota uncouple the function of the Gut-Skin-Brain Axis which is evidenced by the development of diseases in humans or its preclinical models. A full comprehension of the involved mechanism, like high oxidative stress, DNA damage, impaired cell signaling, and associated dysbiosis, will open the way to offer alternative healing methods to drugs but above all, the possibility to prevent the onset of the associated diseases through a correct lifestyle and diet; all in all with the ultimate goal to promote patient's health.

## Author contributions

AF wrote the introduction and the conclusion. All authors summarized the contributions and added comments to the cited papers. All authors approved the submitted version.

## References

[1] Mahmud RM Akter S Khanam Tamanna S Mazumder L Esti I Z Banerjee S. Impact of gut microbiome on skin health: gut-skin axis observed through the lenses of therapeutics and skin diseases. Gut Microbes. (2022) 14:e2096995. 10.1080/19490976.2022.2096995 35866234 PMC9311318

[2] Schiopu CG Stefanescu C Bolos A Diaconescu S Gilca-Blanariu GE Stefanescu G. Functional gastrointestinal disorders with psychiatric symptoms: involvement of the microbiome–gut–brain axis in the pathophysiology and case management. Microorganisms. (2022) 10:2199. 10.3390/microorganisms10112199 36363791 PMC9694215

